# Familial occurrence of Danish and Dutch cases of the bovine brachyspina syndrome

**DOI:** 10.1186/1746-6148-3-8

**Published:** 2007-05-08

**Authors:** Jørgen S Agerholm, Klaas Peperkamp

**Affiliations:** 1 Department of Veterinary Pathobiology, Faculty of Life Sciences, University of Copenhagen, Ridebanevej 3, DK-1870 Frederiksberg C, Denmark; 2 Animal Health Service GD Deventer, Arnsbergstraat 7, 7418 EZ Deventer, The Netherlands

## Abstract

**Background:**

The bovine brachyspina syndrome is a recently reported malformation in the Holstein breed. The aetiology of this syndrome is unknown, but its occurrence following breeding between genetically related and phenotypically normal cattle may indicate that it is an autosomal recessively inherited disorder. Three cases are reported and compared to the originally reported case.

**Case presentation:**

Two Danish cases and a Dutch case are described. The calves were delivered following a slightly prolonged gestation period. Gross lesions consisted of growth retardation, significant shortening of the entire spine and long and slender limbs. Additionally, inferior brachygnatism and defects of several internal organs were recorded. The cases were diagnosed as having the brachyspina syndrome based on the presence of essential lesions. The parents of each case were genetically related and linked to the first reported case by a common ancestor.

**Conclusion:**

The findings support the hypothesis that the brachyspina syndrome in Holstein cattle is inherited autosomal recessively and illustrate some of the assumed phenotypical variation of this syndrome. The brachyspina syndrome may be an emerging disease in the Holstein breed.

## Background

Recently, a new congenital lethal syndrome was reported in a Danish Holstein calf (case DK1) [1]. The syndrome was characterized by severely reduced body weight, widespread vertebral malformation causing significant shortening of the spine (brachyspina), and malformation of the heart, kidneys and testicles. The cause of the syndrome was not determined. But as the calf was the result of artificial breeding between genetically related and phenotypically normal animals and as it was the only affected calf occurring in a herd of 96 adult females, such an incident could be explained by transmission by descent of a defective recessive allele from a common ancestor. However, further cases were needed to evaluate the hypothesis of autosomal recessive inheritance.

Here, we report two additional cases from Denmark (cases DK2 and DK3) obtained through the Danish Bovine Genetic Disease Programme [2] and a case from the Netherlands (case NLD1) submitted for routine diagnostic.

## Case presentation

### Case DK2

**Figure 1:**
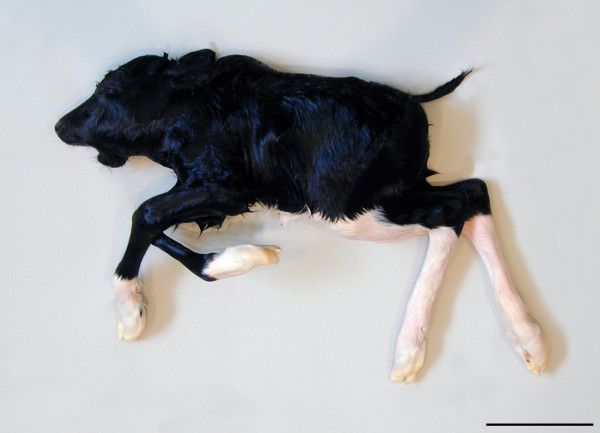
**Bovine brachyspina syndrome in a Danish Holstein calf (case DK2)**. The calf is growth retarded with shortening of the spine and prominence of the thoracic spinous processes. Notice inferior brachygnatism and long slender legs. Bar = 15 cm.

**Figure 2:**
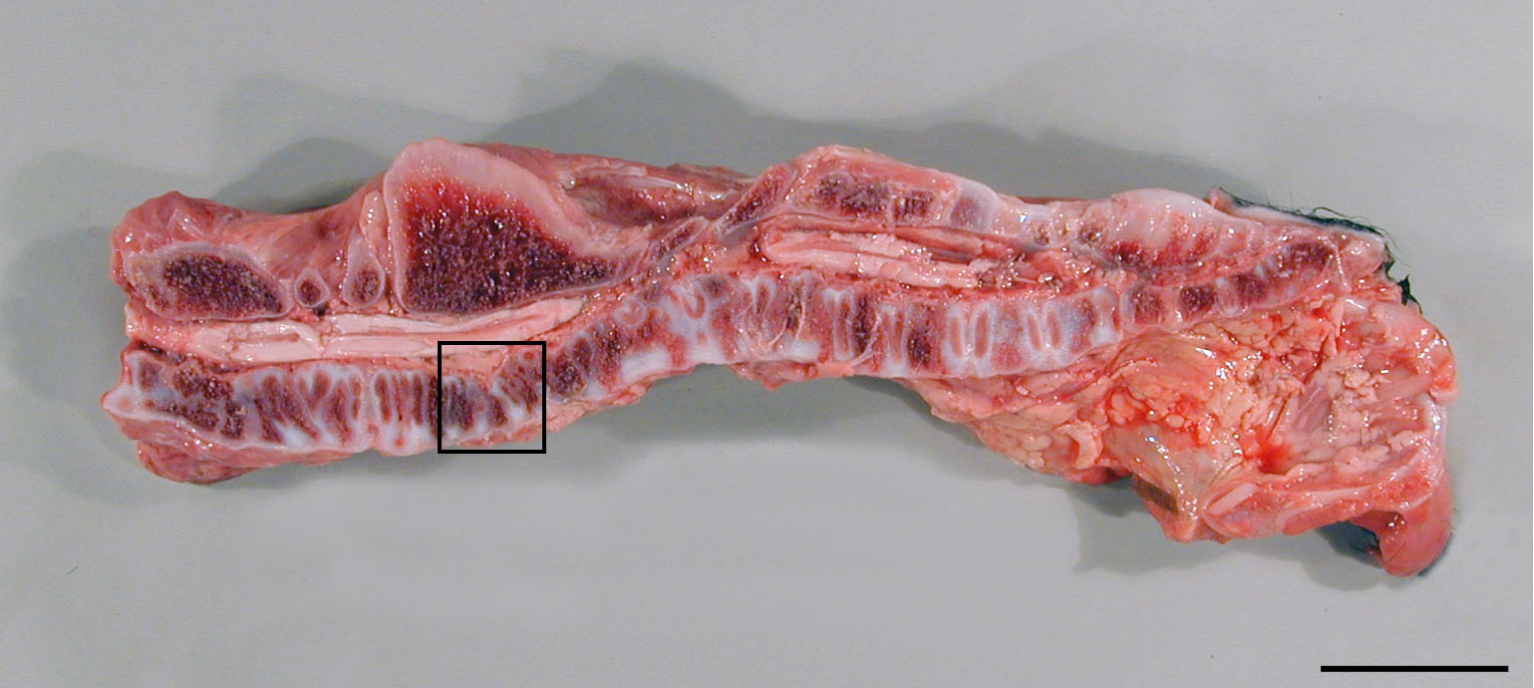
**Widespread malformation of vertebrae in a case of the brachyspina syndrome (DK2)**. Widespread malformation and disorganisation of cervical, thoracic and lumbar vertebrae. Notice the fusion of the spinous processes of thoracic vertebrae. The thoraco-lumbar junction is not cut in midline due to the presence of a scoliosis. The framed area is shown in detail in Figure 3. Bar = 5 cm.

A female Holstein calf with a body weight of 10.8 kg (Fig. 1) was born in March 2006 at gestation day 289 by a two-year-old heifer. The length of the spine was severely reduced with prominence of the anterior part of the thoracic spine and scoliosis at the thoraco-lumbar junction and of the caudal vertebrae. Longitudinal sawing of the spine showed severe disorganisation and malformation of almost all vertebrae, except for the most posterior vertebrae. The anterior thoracic spinous processes had synostosis and increased height (Fig. 2). The head was malformed due to severe inferior brachygnatism and slight caudal displacement of the ears. Longitudinal sectioning of the head demonstrated caudal dislocation and compression of the cerebellum with slight herniation of the cerebellum and medulla oblongata through foramen magnum. The limbs were long and slender with slight symmetric flexion of the elbow and carpus. The digits were slightly rotated laterally.

The volume of the thoracic cavity was reduced and almost occupied by an enlarged heart. The ribs radiated from the spine with non-parallel intercostal spaces. The number of ribs was reduced to 11 in each side, but they were difficult to separate due to the presence of extensive synostoses. The lungs had congenital diffuse atelectasis, were of reduced size and compressed by the heart. Low-grade hydrothorax and hydropericardium was found. The heart had a round shape due to dilation of the right ventricle and hypertrophy of the corresponding myocardium. A high interventricular septal defect was present. Truncus pulmonalis and aorta arose separately from the right ventricle (dextroposition of the aorta). The basis of aorta was dilated.

The volume of the abdominal cavity was also reduced. The anterior part was occupied by an enlarged liver. Colon descendens had agenesia at the entrance to the pelvic cavity and a stenosis approximately 2 cm orally to the agenesia. The prestenotic segments were dilated due to accumulation of meconium, while the poststenotic segments only contained mucus. The kidneys had bilateral hypoplasia with a length of approximately 3 cm. The right uterine horn was hypoplastic. The adrenal glands and ovaries were present at their normal position.

**Figure 3:**
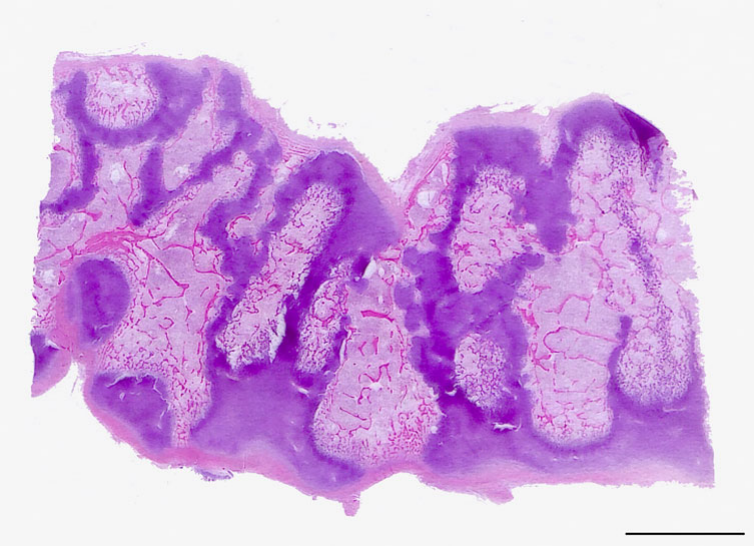
**Photomicrograph of an area of the thoracic spine showing severely disturbed organisation of vertebrae**. Irregular ossified areas are separated by cores of cartilage omitting identification of individual vertebrae. Haematoxylin and eosin. Bar = 5 mm.

Multiple tissues were taken for histopathology and processed as previously reported [3]. The tissues were stained by haematoxylin and eosin. Selected sections were additionally stained by Masson's trichrome connective tissue stain, Van Gieson's method for collagen fibres, and Perls' iron stain. Most vertebral segments showed lack of organisation with irregular interweaving cartilaginous cores enclosing ossified areas and absence of intervertebral discs thus omitting identification of individual vertebral bodies (Fig. 3). The degree of calcification of trabecules varied as some were completely ossified while others had a central core of cartilage. Few individual vertebrae were observed in the lumbar and sacral region (Fig. 2). These vertebrae were separated by dysplastic intervertebral discs and had broad irregular epiphyseal growth zones. Organisation and ossification of the appendicular skeleton were normal. The liver had centrolobular congestion with dilation of sinusoides, atrophy and loss of hepatocytes, mild centrolobular fibrosis, mild haemosiderosis, mild bile congestion and hyperplasia of bile ducts. The kidneys were dysplastic with uneven development of tubules, luminal crystals and periductular fibrosis.

The calf was tested negative for bovine virus diarrhea virus (BVDV) by cell culture and for antibodies against BVDV by an ELISA technique [4].

**Figure 4:**
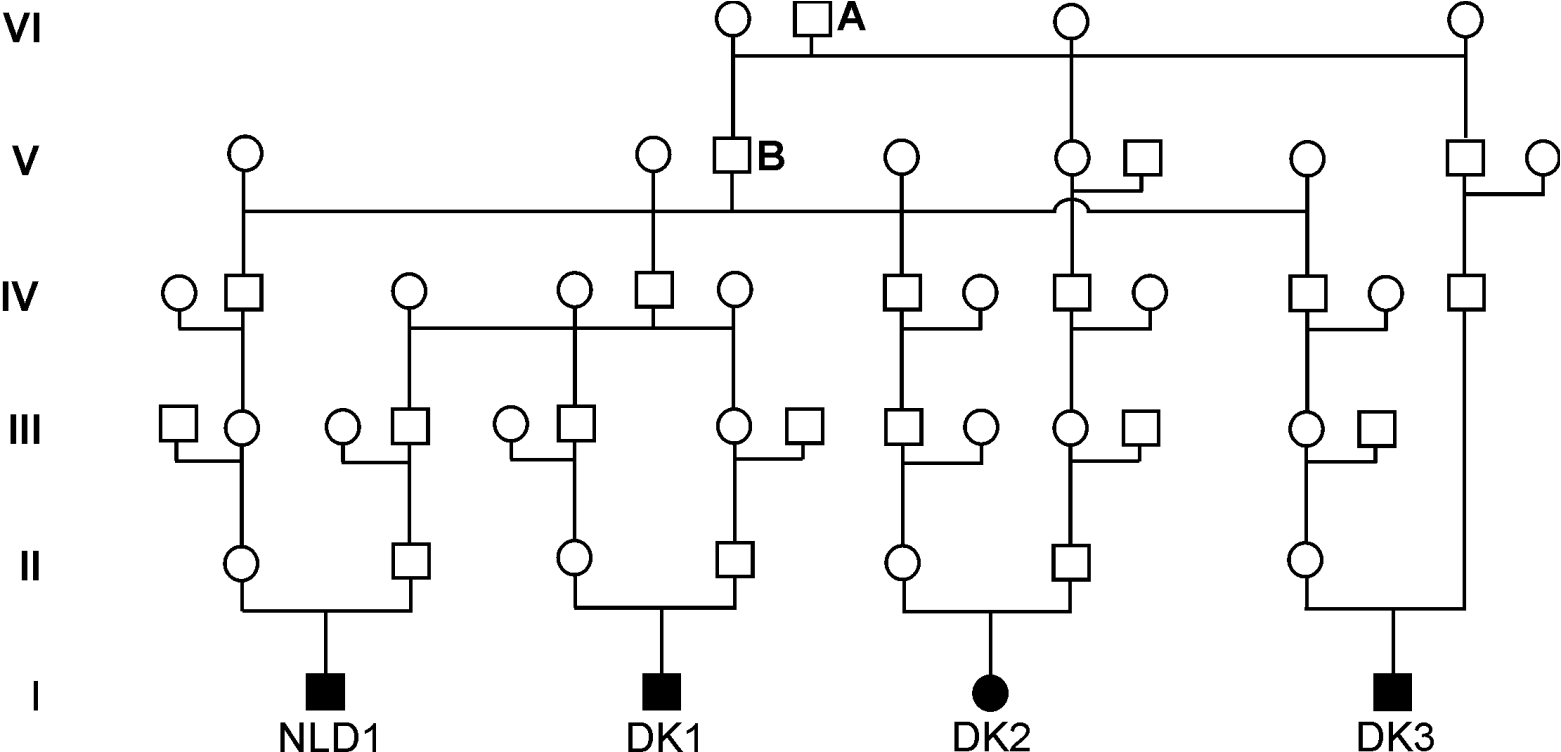
**Genealogic diagram showing the genetic relationship between four cases of the brachyspina syndrome**. Three Danish cases of the bovine brachyspina syndrome (DK1–3) and a Dutch case (NLD1) are genetically related within 6 generations. Each affected calf is the result of breeding between genetically related animals and all parents are related to a common ancestor (A) in generation VI, often through his son (B). □ = phenotypically normal male; ○ = phenotypically normal female; ■ = affected male; ● = affected female.

Examination of six-generation pedigree showed that the parents were genetically related through a common ancestor to the parents of the previously observed case (DK1) (Fig. 4).

### Case DK3

**Figure 5:**
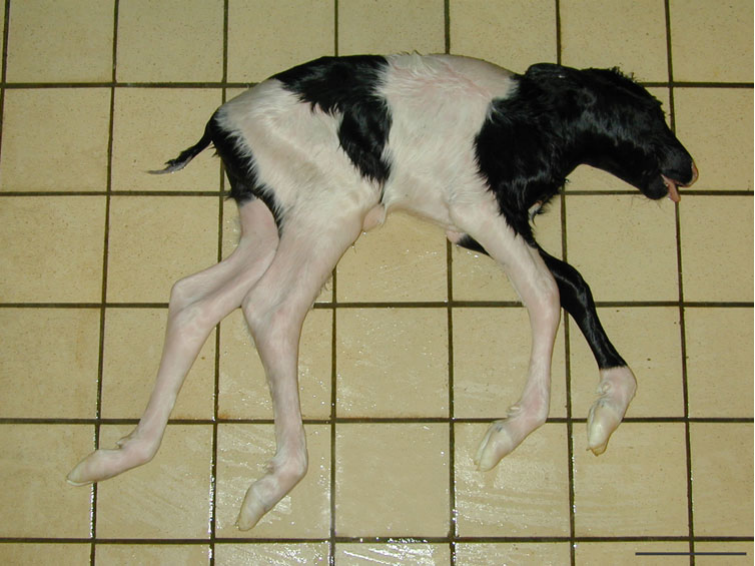
**Bovine brachyspina syndrome in a Danish Holstein calf (case DK3)**. The calf has reduced body weight and significant shortening of the spine. Additionally, a mild inferior brachygnatism is present and the legs are long and slender. Bar = 15 cm.

This case was obtained retrospectively from the archive at the Department of Veterinary Pathobiology. This Holstein male calf was delivered stillborn in February 2003 at gestation day 299 by a two-year-old heifer. The calf had a body weight of 13.6 kg, mild inferior brachygnatism, and severely reduced length of the spine (Fig. 5) due to widespread malformation and synostosis of vertebrae. Atresia of the spiral loop of the ascending colon with prestenotic dilation and poststenotic hypoplasia was present. The kidneys and testes were not found at necropsy. The calf was not examined by histopathology.

The calf was examined for BVDV and foetal antibodies with negative results. The calf was genotyped for the V180F mutation in the *SLC35A3 *gene, which causes the complex vertebral malformation syndrome (CVM) in Holsteins, and found homozygous normal.

The pedigree was compared to pedigrees of the two other Danish cases. The parents were genetically related to the other cases (Fig. 4).

### Case NLD1

**Figure 6:**
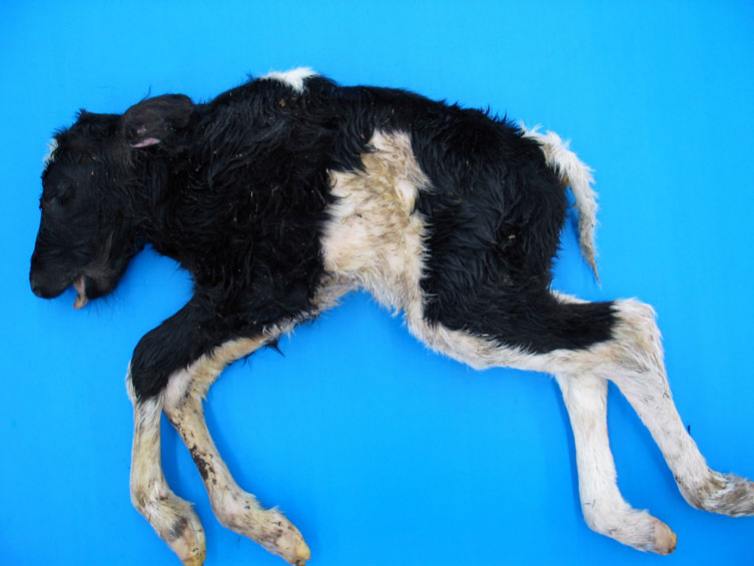
**Bovine brachyspina syndrome in a Dutch Holstein calf (case NLD1)**. The calf has significant shortening of the spine and prominence of the thoracic spinous processes. The body weight is reduced to 10.5 kg. Inferior brachygnatism is present.

A stillborn male Holstein calf with a body weight of 10.5 kg was delivered in August 2006 at gestation day 288 by a 2-year-old heifer. The calf was severely growth retarded with significant shortening of the crown-rump length, thoracic kyphosis, and relatively long thin limbs. The length of the mandible was reduced (Fig. 6). Longitudinal sawing of the spine demonstrated widespread malformation and disorganisation of vertebral bodies. The epiphyseal ossification centres were surrounded by a broad zone of cartilage and the epiphyses of adjacent vertebral bodies were mostly separated by a rudimentary intervertebral disc or completely fused. Cartilagenous peaks often protruded into the diaphyses from the ventral or dorsal margin giving the diaphyses an irregular border and uneven size throughout the entire spine. Internal organ malformations consisted of a high interventricular septal defect, myocardial hypertrophy and complete fusion of the truncus pulmonalis and aorta, reduced size of the kidneys and the left testicle. The right testicle was not found.

Multiple tissues were taken for histopathology, processed by routine laboratory methods and stained by haematoxylin and eosin. Bone tissues were decalcified by Osteomoll^® ^(Merck KGaA, Darmstadt, Germany) prior to processing. Vertebral lesions were characterized by widespread disturbed segmentation and organisation. Intervertebral disc development was mostly absent although protrusion of collagen from the periphery into the epiphyseal anlage was occasionally present. Consequently, adjacent vertebrae often share a common epiphysis with only one ossification centre. The growth zones were highly irregular towards the diaphyses with frequent abnormal arrangement of chondrocytes. The diaphyses were in some areas intersected by cartilaginous cores dividing the spongiosa into inlets of spongious bone. The appendicular skeleton was not examined. The kidneys were dysplastic with immature tubules, dilated tubules, intraluminal crystals and interstitial fibrosis.

The calf tested negative for BVDV by an antigen ELISA technique (HerdChek BVDV Antigen ELISA – Leukocyte, IDEXX Laboratories, Inc., Westbrook, ME, USA). Genotyping for the V180F mutation in the *SLC35A3 *gene was performed after retrieving DNA from formalin fixed and paraffin embedded liver and heart tissue [5]. The calf was genotyped as homozygous normal.

Pedigree analysis demonstrated that the parents of the Dutch case were genetically related to the three Danish cases (Fig. 4).

## Conclusion

The gross morphology of the three cases (DK2, DK3 and NLD1) was in many aspects similar to the lesions found in the original case (DK1) [1] and they were consequently considered to have the brachyspina syndrome. All cases were stillborn and were delivered after a slightly prolonged gestation period. The calves were severely growth retarded, had a significant shortening of the entire vertebral column, and the limbs appeared relatively long and slender (Figs. 1, 5 and 6). Additionally, multiple defects of the internal organs were found, i.e. renal dysplasia and intestinal atresia. However, discrepancies between the cases and the original case were also observed as they all had brachygnatism and one case had brain displacement – lesions that were absent in the first case. Histopathology was only performed in two cases (DK2 and NLD1). Bone lesions in these cases differed from the original case as the development of vertebrae was more disturbed and as the ossification process of the appendicular skeleton appeared normal in case DK2 compared to case DK1. The appendicular skeleton of case NLD1 was not examined. It could be objected that the four calves represented more than one syndrome, as the morphology was not completely identical. This point of view cannot be completely disproved, but it is not surprising that a certain variation is seen. Studies of CVM in Holstein cattle, which is due to a single base mutation [6], have demonstrated that phenotypical variation occurs. This variation is expressed in the severity of vertebral malformation as well as in the prevalence and type of internal organ defects [7,8]. Phenotypical variation is also seen in other skeletal malformations due to single genes, i.e. syndactylism and chondrodysplasia [9,10]. It is possible that a certain morphological variation of the brachyspina syndrome, which could explain the observed differences, could occur as well. At present, a diagnosis of the brachyspina syndrome should be based on the general appearance of abnormal calves (Figs. 1, 5 and 6). A detailed examination of future cases should be performed so that the variation of the syndrome can be determined and DNA should be stored to establish an exact diagnosis if a genotyping test becomes accessible.

The brachyspina syndrome and CVM are malformations occurring in the Holstein breed. Although these syndromes share several features as vertebral malformation, growth retardation and internal organ malformation, discrimination between these defects can be done morphologically. Calves suffering from the brachyspina syndrome have a mean body weight of 10.3 kg compared to a mean body weight of around 25 kg in CVM-affected calves [8]. Vertebral lesions are more widespread and severe in the brachyspina syndrome than seen in CVM, where lesions mostly are restricted to the posterior cervical and the thoracic vertebrae. Consequently, the entire spine is reduced in length in the brachyspina syndrome compared to shortening of the cervical and thoracic spine in CVM. Renal dysplasia, which occurs in the brachyspina syndrome but not in CVM, also seems to be an important diagnostic mark. Finally, CVM-affected calves generally have a slightly reduced gestation period, while calves with the brachyspina syndrome seem to have a prolonged gestation period [7,8]. Definitive discrimination between the defects can be done by genotyping. Genotyping of two cases included in this report and the original case DK1 (Agerholm, unpublished results) separated the two disorders without doubt.

The genealogical examination showed that the cases occurred in a familial pattern and that the parents of each calf were genetically related. A common ancestor to all parents (sire A) was found in generation VI (Fig. 4). The occurrence of the defective calves might therefore be explained by transmission of recessive alleles from this sire to the calves. Sire A and his son (sire B) are widely used US Holstein sires and it is therefore possible that they occur in the pedigree of the parents by chance. It has previously been reported that widely used sires may occur as common ancestors in the pedigree of malformed Holstein calves fortuitously [7,8]. Consequently the observation cannot be regarded as a distinct proof for an inherited aetiology of the brachyspina syndrome and the sire cannot at present be regarded as a carrier of the defect. However, the observations support the hypothesis of a genetic basis of the brachyspina syndrome as the cases occurred in a familial pattern consistent with autosomal recessive inheritance. It is recommended to perform a breeding trial to determine the inheritance of this syndrome and veterinarians are encouraged to report additional cases. Evaluation of breeding data for heterozygous sires used for artificial insemination should be performed when sufficient data are available to evaluate if the brachyspina syndrome is associated with increased embryonic and foetal mortality as for CVM [11]. The brachyspina syndrome may be an emerging disease in the Holstein breed worldwide, if the syndrome is inherited autosomal recessively.

## Authors' contributions

JSA performed the examination of the Danish cases and drafted the manuscript. KP examined the Dutch case and participated in the drafting. Both authors read and approved the final manuscript.
